# Understanding Black Women’s Lived Experiences of Medical Mistrust and Trust in the Reproductive Care Setting: A Nested Sequential Explanatory Mixed Methods Study

**DOI:** 10.21203/rs.3.rs-9889877/v1

**Published:** 2026-07-10

**Authors:** Karen Awura-Adjoa Ronke Coker, Tyler S. Nesbit, Brittney N. Dixon-Daniel, Arie Hendrik Havelaar, Nancy Hunt, Sarah Milbrandt, Adetola F. Louis-Jacques, Larry Forthun, Sarah L. McKune

**Affiliations:** University of Florida; University of Florida; University of Florida; University of Florida; University of Florida; University of Florida; University of Florida; University of Florida; University of Florida

**Keywords:** Medical Mistrust, Trust-building, Patient-Provider, Black Women, Reproductive Health, Mixed Methods, Lived Experience

## Abstract

**Background:**

Medical mistrust inhibits positive healthcare-seeking behavior, resulting in negative health outcomes. We assessed Black women’s medical mistrust and concepts of trust-building provider characteristics in the reproductive health setting in Alachua County, Florida.

**Methods:**

A nested, sequential, explanatory mixed-methods design was employed. In addition, an Integrated Black Women’s Reproductive Health Social Ecological Model (IBWRH-SEM) was developed to construct the survey, interview tools, participant engagement, data collection, and analysis. A sample of 255 self-identified Black women aged 18 and older completed an online survey, with a subsample of 16 completing semi-structured follow-up interviews.

**Results:**

The survey findings highlighted higher mistrust amongst younger, married, and women seeking both allopathic and holistic care. Women who sought both allopathic and holistic settings experienced higher mistrust, lack of support, suspicion, and higher health disparity scores. Also, younger women, women without children experienced lack of support. In addition, findings showed that younger women, women possessing a graduate/professional level degree had higher suspicion and higher health disparity scores. Women who did not have children had higher health disparity score. Furthermore, semi-structured interviews revealed five key trust-building characteristics: transparency, humanizing interactions, comfort and provider tone, acknowledgment of patient insight, and time spent with the patient. These characteristics were essential for trust-building during patient-provider interactions in the reproductive healthcare setting.

**Conclusions:**

Findings suggest structural changes to improve institutional systems and interpersonal provider characteristics in the reproductive healthcare setting. Intervention strategies need to center Black women, so they are supported and, over time, unburdened of the need to self-advocate and vigilant.

## 1. BACKGROUND

“trust feels joyful. … I don’t get nervous when I go see her. I don’t… be scared.”

– (Interview-5) (34 yrs old, not married, college, no children)

Black women are burdened by negative exposure pathways, such as structural racism and implicit bias leading to mistrust of the medical establishment, which impedes their engagement and satisfaction with medical care.^1^ Heightened exposure to discrimination, and negative stereotyping by health care providers increases risk and is a driver of medical mistrust, resulting in adverse reproductive health outcomes including elevated rates of maternal and infant mortality among Black women.^1,2,3^ Dorothy Roberts emphasized that these negative exposure pathways devalue Black women’s bodies and reproductive rights in her book *Killing the Black Body*.^4^ In particular, Black women’s bodies were subjects for reproductive scientific exploitation. James Marion Sims, considered the “father of gynecology,” used the bodies of enslaved women without consent for “medical knowledge construction”, “expansion”, and “surgical assistance”, as he sought to develop the field that continues to be a place of unwarranted harm towards Black women today.^7^ This legacy has contributed to Black women’s current questioning, hesitancy, and medical mistrust.^3,5,6,7,8^ Sims’ contributed to the dehumanizing myth that enslaved Black women had a higher tolerance for pain than white women, which has resulted in the development of racist medical care constructs.^1,9,10^ These constructs have contributed to Black women’s exposure to discriminatory practices and attitudes while seeking care, which exacerbates medical mistrust.^11^

Previous studies have found that, compared to white adults, Black adults report greater group-based medical mistrust.^12,13^ Medical mistrust has been associated with poorer outcomes for people with chronic disease and identified as a pathway by which racism drives health inequities.^11^ Higher levels of medical mistrust have been documented among Black women seeking cervical cancer screening, preventative breast cancer care, and HIV-related care services.^14,15,16^ Black adolescents with poorly controlled asthma and Black sexual minority men have also reported elevated medical mistrust,^11,17^ as have Black African immigrant and refugee communities in the context of genetic testing.^18^ To our knowledge, in the reproductive health context, only one study has assessed group-based medical mistrust among women of color (Black Hispanic/Latinx women, non-Hispanic/Latinx Black women, non-Black Hispanic/Latinx women, and Asian women), particularly for Chronic Vulvovaginal Pain (CVVP).^19^ Researchers assessed only one of three Groub Based Medical Mistrust (GBMMS) subscales, not the whole scale, and found variation in suspicion across all racial and ethnic groups. To our knowledge, the Group-Based Medical Mistrust Scale (GBMMS) has not been used to assess group-based medical mistrust and conceptualizations of trust *in provider-patient interactions in the context of reproductive healthcare* for Black women. Thus, one objective of this study was to psychometrically validate the GBMMS, ensuring the scale’s appropriateness for our sample.

An increasing number of studies have prioritized the inclusion of lived experiences to generate solutions to reproductive and health inequities.^3,5,20,21,22^ To forge long-lasting changes to these outcomes, Crear-Perry et al. have called on researchers and scholars to center the experiences of those communities most affected. Specifically, they note that Black women hold answers to what respectful, trustworthy, and equitable reproductive care might look like, ensuring optimal well-being and spaces to thrive. This study acknowledges the years of community organizing and the collective work of organizations such as the Black Women’s Health Imperative and many others in implementing reproductive health solutions for Black women.^20,21,23,24,25,26,27,28,29^ Thus, a second objective of this study was to work with Black women to document the range of ways that they understood and experienced mistrust and trust towards their reproductive health provider. We achieved both objectives by first assessing medical mistrust and later discussing with women their ideas of trust-building characteristics in a provider.

## 2. METHODS

**Figure 1:**
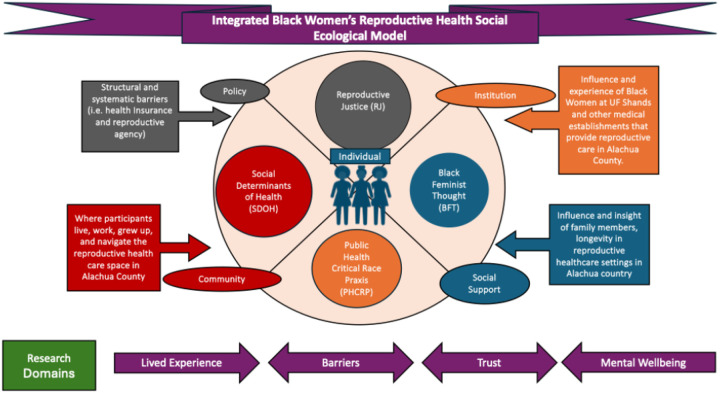
Integrated Black Women’s Reproductive Health Social Ecological Model (IBWRH-SEM) Theoretical Framework

A two-phase, nested, sequential, explanatory mixed-methods design guided this study. Using this design, data were collected to ascertain the lived experience of mistrust and trust in the reproductive care setting of Black women. Phase one (quantitative) utilized an online survey, followed by phase two (qualitative), which utilized semi-structured interviews with a subset of survey participants. Refer to (supplemental Figure A.1) for the research flow chart. We developed a theoretical framework to better understand the lived experiences of participants, the Integrated Black Women’s Reproductive Health Social Ecological Model (IBWRH-SEM) (Fig. 1) was developed to construct the survey, interview tools, participant engagement, data collection, and analysis.

### 2.1. Theoretical Framework

The IBWRH-SEM includes four heuristics from scholars and Black theorists across different disciplines. As visualized in Fig. 1, the four heuristics are integrated within the socio-ecological model (SEM), each representing a theoretical lens utilized within these four levels of inquiry (policy, institutional, community, and social support) in interpreting participants’ responses concerning mistrust and trust. The intersecting research domains are represented at the bottom of the schematic. The first heuristic is the Reproductive Justice (RJ)^32^ lens utilized at the policy level to frame the influence and impact of structural and systemic barriers in accessing reproductive care. In addition, this lens supports a more nuanced analysis of the lived experiences of mistrust and trust. It helps understand predictors of mistrust and the characteristics of trust. The social determinants of health (SDOH)^21,33^ provides a second lens, used to interpret the implications of barriers due to structural and environmental influences on mistrust and trust. The public health critical race praxis (PHCRP)^34^ lens facilitates an understanding of the present state of healthcare systems, research, and engagement with participant experiences in medical establishments. Finally, the Black Feminist Thought (BFT)^35^ provides the capacity to interpret participants’ experiences and narratives about themselves and other Black women in their lives. BFT enables the interpretation of mistrust and trust outcomes across participants’ diverse reproductive lifespan and patient-provider interactions. Overall, IBWRH-SEM provides this study with a pathway for clarity, understanding, inclusion, and interpretation of Black women’s lived experiences of trust and mistrust.

### 2.2. Quantitative phase

Demographic data and measurement of medical mistrust were collected from March to September 2022 using REDCap.^30,31^Participants received $10 compensation for participation. Participants were individuals living in Alachua County, Florida who self-identified as Black Women, were 18 years or older, and/or were pregnant or a mother of young children (5 years or younger). Recruitment used a two-armed approach: institutional research databases and community engagement, leveraged by a Community Advisory Board (CAB) and HealthStreet, a community outreach center housed within University of Florida’s Clinical and Translational Science Institute (CTSI).

### 2.3. Qualitative phase

Overall, 16 participants participated in 9 interviews (as noted above, participants were from the larger survey sample). For their participation, they received $30. The research team conducted a thematic analysis of the one-on-one, paired, and group interviews in Atlas.ti, with a set of analysis guidelines developed by the research lead using a six-step process.^45,46^ The coding process is described in (supplemental Table A.2).

### 2.4. Measures

The Group-Based Medical Mistrust Scale (GBMMS) 12-item assesses the tendency to distrust those who do not belong to one’s ethnic group and/or distrust systems that do not represent one’s ethnic group based upon a legacy of racism or unfair treatment.^36^ In addition to assessing overall GBMMS, we separately calculated its three subscales: suspicion (SUS), lack of support (LOS), and group disparities in health care (DISP).^36^

### 2.5. Healthcare Setting

We defined the reproductive care space in this study as the setting in which patient-provider interactions occur regarding comprehensive services encompassing fertility, family planning, pregnancy- and maternal-related needs, and post-partum care. An allopathic setting as a system in which medical doctors and other healthcare professionals (such as nurses, pharmacists, and therapists) treat symptoms and diseases using drugs, radiation, or surgery. A holistic setting as one where the form of care considers the whole person -- body, mind, spirit, and emotions -- in the quest for optimal health and wellness. The outcome indicating “both” on the survey meant participants used both allopathic and holistic settings defined above.

### 2.6. Data Analysis

Survey analysis was conducted in R (R Core Team 2023) using descriptive statistics and multivariate linear regression modeling.^47^ Univariate analysis was utilized to determine significant independent predictors with a p-value < 0.20 and q-value < 0.20, refer to (supplemental Table A.1). Stepwise forward regression was used to fit the final model by entering predictor variables based on p-values in a stepwise manner. Model assumptions were assessed using diagnostic plots, and multicollinearity was assessed using the variance inflation factor.

Qualitative analysis of sixteen participant semi-structured interviews (SSI) consisted of a team of two research assistants and the two PI’s (first and second author) transcribing, coding, and thematic analysis in ATLAS.ti (23.3.1)^37,45,46,47^desktop for Mac, refer to (supplemental Table A.2) for further details on the process. SII occurred from October 2022 to April 2023. An integrated and rationale table of the relevant survey findings and interview questions and interview guide can be found in (supplemental Table A.3).

## 3. RESULTS

The demographic characteristics of the 255 survey participants and 16 interview participants are presented in Table 1. The mean age of the surveyed participants was 34 years. About 51% of participants sought provider care in an allopathic setting, with 27% in a holistic care setting, and 22% in both settings. Among survey participants, a majority (62%) were married. In terms of education, 34% had a GED/high school diploma and 37% had a college degree. About 28% of participants’ annual income was $50,000-%75,000 a year, 67% had private health insurance, 44% utilized a women’s health clinic for their reproductive healthcare needs, 47% indicated having children, while 41% indicated being pregnant. Most indicated awareness of midwifery (76%) and doula (70%) services in Alachua County. The mean age for interviewee’s were 38 years, while 24% were married, 34% had a college degree, 41% had a graduate and professional degree, 75% utilized a hospital or women’s health clinic and 63% had children.

We compared the interview subsample (n = 16) to the survey respondents (n = 255) using Fisher’s exact tests, results presented in (supplemental Table A.4). Interview participants differed in marital status (p = 0.003; fewer married), pregnancy status (p = 0.006; fewer currently pregnant), healthcare setting (p = 0.018; greater recruitment from hospital/Women’s health clinics), and annual income (p = 0.012; more ‘Other’ income and fewer >$75k). Education also differed (p = 0.035), though this was borderline after false discovery rate adjustment. No significant differences were observed for provider type, insurance, having children, or awareness of midwifery/doula services (all p ≥ 0.12).

### 3.1. Group-Based Medical Mistrust

Confirmatory factor analysis (CFA) of the GBMMS was conducted to validate the psychometric properties for the study context and population. CFA results supported the original three-factor model;^36^ the model fit the data well, as all the fit indices met their thresholds, [X^2^= 97(p-value = 0.00), the Root Mean Square Error of Approximation (RMSEA) = 0.06 (90% CL: 0.04 – .08) and the Standardized Root Mean Square Residual (SRMR) = 0.06].

The percentage of participants in agreement with the GBMMS items and mean scores can be found in Table 2. About a quarter of participants indicated that in their reproductive healthcare setting, they believed that their healthcare providers did not take the medical complaints of patients in their ethnic group seriously. Participants indicated that they had personally been treated poorly or unfairly by reproductive healthcare providers because of their ethnicity. The score range for GBMMS is 12 (lowest mistrust score) to 60 (highest mistrust). The total GBMMS mean score was 32.9 (4.9), indicating that participants, on average, express moderate mistrust below the overall scale midpoint, with subscale scores ranging from 9.7 to 16. Interview participants had comparable scores, though the subsample is not representative of the larger survey sample found in Table 2.

A stepwise multiple regression analysis was conducted. As detailed in Table 3, age was significantly associated with the total GBMMS score and all three subscales. An increase in age by one year was associated with a 0.15-point lower GBMMS score, meaning lower mistrust. Participants who used both a holistic and allopathic setting had a 1.93-point increase in the GBMMS score, indicating higher mistrust compared to individuals who used just an allopathic setting. On average, unmarried women (e.g., engaged, separated, divorced, widowed, or never married) had a 2.3-point decrease in GBMMS compared to married women, indicating higher mistrust amongst married women.

An increase in age by 1 year was associated with a 0.06-point decrease in the LOS score for women, indicating older women experienced slightly more support from their reproductive care provider. Women who reported having children had a 0.90-point decrease in the LOS score, indicating they experienced slightly more support during care than non-parents. On average, women with public health insurance had a 1.3-point decrease in LOS compared to women with private insurance. Participants who utilized both a holistic and allopathic setting had a 1.1-point increase in LOS compared to women who only utilized an allopathic setting.

An increase in age by one year was again associated with a 0.19-point decrease in SUS score. At the same time, those with a graduate or professional degree had a 2-point increase in the SUS score. Participants who used only a holistic setting had a 1.3-point increase in SUS compared to those who used only an allopathic setting. Those who used both a holistic and allopathic setting also had and 1.8-point increase in SUS compared to women who only utilized an allopathic setting.

An increase in age by one year was also associated with a 0.06-point decrease in DISP. Those with a graduate-level or professional (e.g., medical school) degree were associated with a 1.7-point increase. Similarly, holding age and education constant, those with children had a 0.79-point decrease in DISP.

## 4. REPRODUCTIVE SPACE AND PATIENT-PROVIDER TRUST-BUILDING CHARACTERISTICS

**Figure 2:**
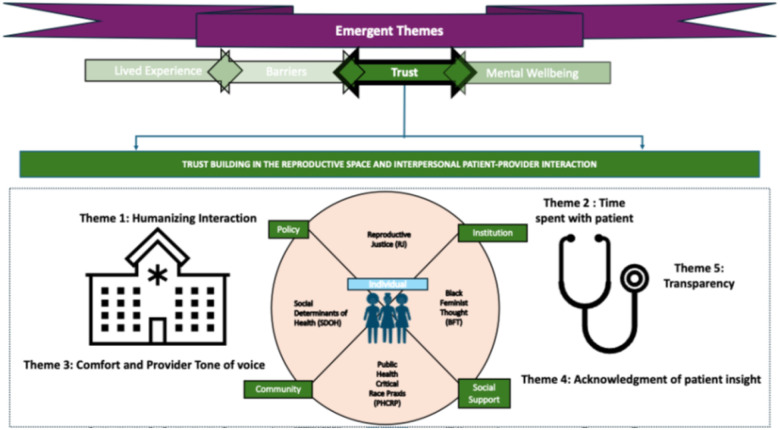
Emergent Themes from participant interviews

The survey results were contextualized in the interviews via the IBWRH-SEM, across the four integrated research domains: lived experiences, barriers, trust and mental wellbeing. Within the trust domain five themes emerged in the context of the cause of mistrust within the reproductive care space and interpersonal behavioral characteristics of providers relevant to trust-building for Black women. We found five emergent themes for trust building in this institutional and interpersonal capacity, including 1) *humanizing interactions*, 2) *time spent with patient*, 3) *comfort and provider tone of voice*, 4) *acknowledgment of patient insight*, and 5) *transparency*. Refer to Fig. 2 for a schematic of the five emergent themes. These thematic findings, each described in more detail below, reflect the perspective of women who likely utilize hospital/clinic settings for reproductive healthcare and have higher educational attainment, e.g. college and graduate/professional degrees.

### Theme 1: Humanizing interaction

For a Black woman seeking reproductive services, a humanizing interaction consists of a provider sitting down, actively listening, asking questions to get to know them as a person, and leading with empathy and care for both their physical and mental well-being. Women reported feeling supported when they are heard without judgment during interactions with their provider. They also stressed the importance of feeling visibly seen, meaning their provider was aware of and respected their presence as a human being. In addition, being heard empowered them during their interactions:

So, if you’re already feeling that you’re not seen or heard, you go to a health care provider, you’re not going to be forthcoming with information, you’re not going to be willing to trust them with providing, you know, with giving you the care that you need.

- (Interview-3) (46yrs old, graduate/professional degree, not married, and no children)

Woman to woman or, you know, human to human sometimes, you know, it’s just like, let me know what this means. Can you explain it to me further? And she does. And she did. And that makes me feel very comfortable. And that makes me trust her. Because I know that she’s just going to talk to me.

- (Interview-7) (28 yrs old, married, college degree, has children)

I feel like trust is trusting someone that’s being humble with you. And trust is respect and understanding and there’s someone that you can, someone that is approachable…And so that, that not only is is not robotic, and not seeing you as just a file in a case where you walk into the office, you know, knowing that you are a person or human being so that so that you could trust them and then they, you know, they can care for you and trust you it goes both ways. So that’s what I feel that trust is…making sure that you can see the individual and, you know, be ready to take in my perspective and include me, and not just see, you know, some information on the paper or on the iPad, but you know, really be able to like to see the whole picture and that that person.

- (Interview − 8) (30 yrs old, not married, GED/HS, no children)

The women represented were in their 20s, 30s, and 40s, with and without children, and had a GED/HS, College, or Graduate/Professional degree. They all reported the importance of been seen, heard, and treated in a humanizing manner, such as when a provider is more relatable, compassionate, and civilized and has humanist qualities, such as kindness and empathy. An example is a provider tailoring the reproductive care environment to better fit their care needs rather than leading with coldness or mechanization care approach.

### Theme 2: Time Spent with Patient

A provider’s capacity in taking the time to understand, empathize, honor, and address concerns, rather than just looking at their charts and rushing through appointments was identified by participants as important. One participant shared that the rush and lack of patience among providers during reproductive services facilitated mistrust. They felt that when the providers lacked empathy due to time constraints, their care was less of a priority. Specifically, due to healthcare system constraints, such as short appointment times, they felt rushed, diluting the care interaction with a provider. Seeking reproductive care services during pre-conception, pregnancy, or post-partum care may be filled with questions, and having the time to discuss is important. This is especially true for Black women, where unfortunate feelings of fear due to historical and current reproductive harm exist. As one participant described:

They’re not listening. They’re rushed… But I think when you become a practitioner in your providing care, you have to be more cognitive of you’re not dealing with numbers or statistics or graphs you’re dealing with flesh, heart, or soul, you know, a person with feelings a person with concerns, a lot of times you’re dealing with a person with fear……..But I would like someone that understand me as a person and when I tell them something that they’re just not there for that 15 minutes of you know, and will also just not just trying to put me on medication or explaining things. So yeah, I need a doctor that actually care about me.

- (Interview − 6) (47yrs old, not married, grad/professional degree, has children)

This woman expressed the negative impact of limited time spent with her provider and the implications on trusting the provider and the care received. By contrast, when a patient experiences a provider who takes and invests time in understanding and addressing their concerns, this provides an important pathway for trust-building rather than erosion.

### Theme 3: Comfort and Provider Tone of Voice

A provider creating a comfortable care space with a positive tone of voice was highlighted as key to establishing trust. These were crucial for patients to feel reassured to confidently entrust their care to a provider. When Black women felt comfortable, they reported easily engaging in conversation and asking questions pertaining to resources to continue seeking reproductive services. Participants described the relationship between comfort and trust in these ways:

Trust makes me feel comfortable.

- (Interview − 2) (26yrs old, not married, grad/professional degree, no children)

So trust is like you walking in, I feel comfortable telling you I’ve got problems with such and such and such…Be whatever it is, I have. And I expect you to deal with my problem and talk to me about the problems that I’m having, you know, in a tone that I can understand.

– (Interview − 9) (68 yrs old, not married, college degree, has children)

Women of different ages expressed that a provider prioritizing their comfort and engaging with a speaking style that is reassuring facilitated trust-building for them.

### Theme 4: Acknowledgment of Patient Insight

Participants discussed the challenges they faced with advocating for themselves, emphasizing the importance of finding providers who understand their viewpoints. Qualities like patience, validating their concerns, and involving them in decision-making about their care led to trusting, meaningful relationships. Participants described this characteristic in these ways:

It makes me excited to just be a part of my care because I trust you, you’re laying everything out for me. So you’re laying everything out for me, you want me to be a part of my care. Like you don’t want to just like hide things from me or keep me in the unknown. Because if you want me to know you that you want me to be a part of what’s going on.

– (Interview − 1) (29 yrs old, married, college, has children)

When I’m with a care provider… even though they might have the textbook, you know, information or the solutions to the things I’m dealing with. I need them to know that I know my body … So I think that’s another aspect of trust.

-(Interview-6) (47yrs old, not married, grad/professional degree, has children)

These women with different demographic characteristics share the similarity of wanting a provider to acknowledge their insights and the role this plays for them in trust-building.

### Theme 5: Transparency

According to participants, establishing trust requires transparency, which entails a provider being honest and effective in their communication with a personable demeanor. A provider sharing information in a straightforward manner was important, as participants described:

For me, trust is just an, like open honesty, and then someone who would do the best for me, who I know is putting their best foot forward and to try to find the answers for my issue. And I would say that’s kind of like and also not hide anything.… You know, tell me what’s going on. And so that way I can adequately prepare instead of like. Oh, oops, by the way, we found this, you know, but if you already had that feeling or concern ahead of time, why didn’t you feel the need to communicate it with me?

– (Interview − 7) (28 yrs old, married, college degree, has children)

Whatever I needed or whatever questions I had, they answered. They answered in a way that I could understand what they were telling me…answers me in a way that I understand. And I’m not illiterate by any means. But I just don’t want you to come to an all those medical terminology, that medical terminology me when a lot of it most people don’t understand…

– (Interview − 9) (68 yrs old, not married, college degree, has children)

These trust-building characterizations were shared from the lived experiences of women seeking fertility, family planning, pregnancy/maternal, and post-partum reproductive services. In entering the reproductive care setting, women wanted time to be a priority and their care not to be rushed, specifically during such an important period seeking various comprehensive reproductive care services. Participants articulated wanting a comfortable setting, where empathy is present, and they are supported and reassured in finding a solution to all issues that they bring to their provider. Lastly, transparency meant the provider communicating honestly and straightforwardly, without hiding any information that could harm the patient. When these key characteristics are embodied in the reproductive care space by a provider, a pathway for trust building was possible. When interactions lacked these characteristics, patients reported that medical mistrust was elevated, as evidenced by increasing barriers to care, lack of adherence to otherwise recommended services, and a negative impact on mental health.

## 5. DISCUSSION

The aim of this study was to assess medical mistrust among Black women toward medical care and to explore how they conceptualize trust and trust-building with providers when seeking reproductive care services. We centered Black women’s lived experiences using our novel IBWRH-SEM framework. IBWRH-SEM reflects their identities and histories and documents health inequities in the context of reproductive healthcare. This framework also provides a structured study design and a pathway to effectively engaging participants, as well as contextualizes the survey and interview outcomes. Its power lies in helping contextualize both the historical background to medical mistrust and the provider characteristics identified as essential for trust-building. This same framework also illuminates why Black women have so often adopted self-protective strategies when navigating reproductive healthcare, which has disproportionately caused more harm than care. As Black women continue to face unequal reproductive health outcomes shaped by policy and diverse barriers (socio-cultural, institutional, and systemic), it must be recognized that mistrust is a rational and reasonable response to marginalization, social and individuated trauma, and racism.^39^ Following the survey, individual and group semi-structured interviews occurred stratified by holistic care, postpartum, older women, nulliparous, and nulligravida. The sequential explanatory design expanded on the specific areas of interest that emerged from the survey results, enabling deeper exploration of participants’ lived experiences with trust and mistrust. As a historically marginalized group, Black women face increased vulnerability, because they are placing trust in entities with power (e.g., health system, providers).^43^ Black women often not by choice, have to self-advocate, be vigilant or self-protective when they seek care.

Findings from our survey and interviews offer new insights into which demographic groups of Black women report higher degrees of medical mistrust in reproductive health care settings, as well as those provider characteristics deemed necessary to nurture or further build trust. Notably, a quarter of participants reported their reproductive care providers did not take their medical complaints seriously. Many reported being treated poorly or unfairly because of their race and ethnicity. These findings align with Cuevas et al.^40^, who found that medical mistrust and perceived discrimination arise when Black women feel that their symptoms are dismissed or that clinicians fail to show them respect. Similarly, Axelrod et al.^41^ reported that Black women often enter obstetric care with a pre-existing mistrust of both providers and the healthcare system, factors exacerbated by insufficient patient-centered and dialogical communication. In sum, these findings suggest that medical mistrust among Black women often emerges as a form of vigilance and self-protection. Mistrust is not a burden marginalized groups like Black women must overcome; rather, it reflects institutional failures that must be addressed.^16^ This form of Black female dignity and assertiveness speaks to institutional and clinical failures, which should be systematically addressed in medical education and programming.

### 5.1. Medical Mistrust in the Reproductive Care Setting

In our multivariate regression analysis, we found that age, provider type, and marital status were significant predictors of higher overall medical mistrust. Younger women had significantly higher medical mistrust compared to older women. This may be that older women visit their reproductive provider less often, as well have an established provider with whom they have established rapport and trust. This finding about higher mistrust amongst younger women is consistent with Cuffee et al.’s finding that younger Black women had higher mistrust in their health care providers due to race and gender-based discrimination.^38^ Our participant Age was also significant across all three medical mistrust subscales. Additionally, we found that younger women felt a lack of support from their providers, suspicion in the reproductive health care space, and faced unequal care treatment during their patient-provider interactions. Women without children were also more likely to feel a lack of support and face unequal care treatment compared to those who had children. This suggests that those seeking care who are child-free or childless face negative reproductive care experiences that may shape mistrust of the setting and provider. Furthermore, women with public health insurance (i.e., Medicaid) also felt a lack of support from their providers, an indication that Black women facing economic disadvantages do not get the support they need. Moreover, women with a graduate or a professional degree were also more likely to be suspicious or express facing unequal care treatment. This suggests that even as a highly educated individual, which may be a contributing factor to higher economic access, Black women continue to face barriers. Our findings are consistent with Adams et al.’s findings that Black women with a college degree or higher had higher mistrust during their patient-provider interactions.^1^ Together, these findings suggest that for various reasons, Black women across myriad lived experiences and reproductive care needs, such as younger women, women without children, and the highly educated, all experience heightened vigilance in their reproductive care encounters. The BFT and RJ frameworks support our understanding of why younger women, college, and professionally educated women are experiencing higher medical mistrust and feeling a lack of support by highlighting the presence of the intersectional identities and barriers these women navigate in the reproductive care setting.

Additionally, we found that women who used both a provider in an allopathic setting and a provider in a holistic setting had higher mistrust, felt a lack of support, and suspicion compared to those who only utilized a provider in an allopathic setting. Women who only used a provider in a holistic setting had higher mistrust as well. To our knowledge, these findings are novel and require further exploration. This suggests that medical mistrust is not solely based on the training or setting of the reproductive provider, but that institutional and interpersonal characteristics matter, regardless of the environment. Future studies should assess the drivers of medical mistrust for individuals in non-allopathic healthcare-seeking settings.

Another notable finding in this study was that being a married woman was significantly associated with higher medical mistrust. This may that married women seek care services more often than unmarried women. To our knowledge, this is a novel finding. Though not representative of our surveyed population, we found that in our interviews, the reproductive care being sought by married women was fertility, pregnancy-related, maternal health, or post-partum. This suggests that married women seeking these services are also having to self-advocate and vigilant when entering these spaces, compared to unmarried women. Further research should explore the cause of medical mistrust amongst married women seeking reproductive care. Specifically, determining what barriers (e.g., social, structural, institutional) may exist, and perhaps the role of their partner’s support as well as opinion when seeking these reproductive care services. Furthermore, assessing the role and presence of partners, including husbands and significant others, and their influence on trust-building may provide insight supportive of interventions centering Black women.

### 5.2. Trust Building

These survey findings identified significant populations of Black women with higher medical mistrust, whom we then interviewed to understand their conceptualization of trust in the reproductive setting. They shared detailed insights about mistrust, lack of support, suspicion, and their unequal experiences. Five core trust-building characteristics were described: 1) having a humanizing interaction; 2) the time spent with the patient; 3) comfort and provider tone of voice; 4) acknowledgment of patient insight; and 5) transparency. Anderson defined overall trust as the “willingness to be vulnerable under conditions of risk and uncertainty”.^42^ When a provider fails to acknowledge a patient’s perspectives, participants highlighted the necessity of self-advocacy due to a lack of support. They struggled during interactions with providers who did not take their concerns seriously. These experiences emphasize the importance of providers trusting a patient’s knowledge of their own body and the need for providers to listen, provide more time, be transparent, and offer personalized care. These characteristics were intertwined throughout our interviews and suggest that Black women don’t want the necessity of self-advocacy when they seek reproductive services. The value and benefit to their overall mental well-being would be significant if changes occurred at the institutional, structural, and provider levels to improve these care interactions. Richmond et al.^43^ write that medical paternalism, racism, and marginalization shape trust, but the few trust frameworks and models developed for health systems fail to depict the influence of these structural factors. Scholars have documented the correlation between structural racism and resulting adverse risks Black women face in the reproductive care setting.^44^ These outcomes mirror our findings on the lack of humanizing care, institutional barriers, and the impact of historical trauma Black bodies have experienced, which has an impact on medical mistrust.^1,4,17,21^

This work aimed to develop and apply a framework that acknowledged the structural factors underlying reproductive health inequities for Black women. We also contribute new insights into which populations of Black women exhibit the highest levels of group-based medical mistrust, and to the growing literature on trust building at the institutional and provider-patient levels. Although medical mistrust has been studied in a variety of healthcare contexts, there is no study to our knowledge that has assessed both Black women’s medical mistrust and conceptualization of trust-building characteristics specific to the reproductive care provider setting.

### 5.3. Study Strengths and Limitations

The strength of this study comes from the lived experiences of Black women. In addition, the IBWRH-SEM provided the ability to engage with individuals, develop the research questions, and interpret the results. Additionally, this study mixed methods and design enabled a cross-cutting exploration of both mistrust and trust-building, providing a pathway to interventions for Black women, providers, institutions, and policies, for real-world impact. Lastly, given the lead investigator’s racial identity, racial concordance influenced Black women’s comfort levels in sharing their experiences and participating in the study.

The study also had limitations. First, the transferability of the qualitative themes is limited to fewer married/pregnant participants and more hospital- and women’s clinic-based users. Second, the study took place in Alachua County, a university town where younger populations may be more engaged in research. Third, the study took place from early 2021 to late 2022, during which early COVID-19 restrictions limited our qualitative phase. The initial research design included a more robust stratification strategy for focus group discussions, but a lack of responsiveness from survey participants required a pivot to conduct in-person and Zoom interviews for the qualitative component.

## 6. CONCLUSION

This study calls attention to key populations of Black women, specifically younger women, educated women, and married women, who face barriers at the institutional, structural, and provider level when seeking reproductive care. This indicates that future interventions should identify which providers Black women trust and how to include Black women in care interactions. For example, trusted stakeholders for Black women seeking reproductive services may include midwives and doulas for pregnant and post-partum women, or older Black women providing support to younger women, married women seeking care. We propose from our findings that because older Black women have less medical mistrust, future interventions can be developed where they are partnered with younger women seeking care. A targeted intervention such as this centers Black women and provides them with a safety net of comfort, ownership, and power, and could influence both interpersonal patient-provider relationships and their health outcomes.

Trust building takes time and genuine intentionality across systems, particularly at the institutional and interpersonal levels. Furthermore, Black women cannot continue with the emotional labor nor be asked to shoulder the burden of patience while systems that have long failed them slowly evolve; the responsibility for change lies with institutions, not the individuals harmed by them.

In summary, we suggest developing interventions that not only center Black women but also target structural barriers to trust at the institutional and interpersonal levels in reproductive care settings. This could improve the reproductive care of younger women, women without children, and married women seeking fertility, family planning, pregnancy, maternal health, and post-partum care.

## Supplementary Files

This is a list of supplementary files associated with this preprint. Click to download.

- supplementaldocumentSSBMC.docx

## Figures and Tables

**Table 1:** Demographic Characteristics of Survey and Interview Participants

Variables	Survey n = 255	Interviews n = 16
	n (%)	n (%)
Age		
Mean (SD)	34 (10)	38 (11)
Provider Type		
Allopathic	130 (51%)	12 (75%)
Holistic	70 (27%)	1 (6.3%)
Both	55 (22%)	3 (19%)
Marital Status		
Married	159 (62%)	4 (25%)
Other	96 (38%)	12 (75%)
Education		
GED/HS	86 (34%)	1 (12%)
College	95 (37%)	6 (35%)
Grad/Prof	55 (22%)	7 (41%)
Other	19 (7.5%)	2 (12%)
Annual Income		
<$30,000/year	41 (16%)	2 (12%)
$30,000–50,000/year	65 (26%)	5 (31%)
$50,000–75,000/year	70 (28%)	3 (19%)
>$75,000/year	63 (25%)	1 (6.3%)
Other	15 (5.9%)	5 (31%)
Insurance		
Private	170 (67%)	10 (63%)
Other	85 (33%)	6 (38%)
Healthcare Setting		
Hospital or Women’s Health Clinic	114 (45%)	12 (75%)
Other	141 (55%)	4 (25%)
Indicated having Children	121 (47%)	10 (63%)
Pregnant	104 (41%)	1 (6.3%)
Aware of Midwifery Services	193 (76%)	13 (81%)
Aware of Doula Services	178 (70%)	9 (56%)

**Table 2:** Percentage of participants in agreement with the GBMMS items (n = 255)

*Suspicion*	Strongly Disagree	Disagree	Neutral	Agree	Strongly Agree
- Item 3	56 (22%)	61 (24%)	83 (33%)	41 (16%)	14 (5.5%)
- Item 4	47 (18%)	70 (27%)	62 (24%)	64 (25%)	12 (4.7%)
- Item 5	73 (29%)	65 (25%)	83 (33%)	25 (9.8%)	9 (3.5%)
- Item 6	66 (26%)	77 (30%)	75 (29%)	30 (12%)	7 (2.7%)
- Item 7	31 (12%)	65 (25%)	71 (28%)	63 (25%)	25 (9.8%)
- Item 9	28 (11%)	54 (21%)	70 (27%)	70 (27%)	33 (13%)
*Group based disparities in healthcare*					
- Item 8	45 (18%)	66 (26%)	81 (32%)	51 (20%)	12 (4.7%)
- Item 10	71 (28%)	75 (29%)	68 (27%)	26 (10%)	15 (5.9%)
- Item 11	63 (25%)	79 (31%)	74 (29%)	24 (9.4%)	15 (5.9%)
*Lack of support from healthcare providers*					
- Item 1	26 (10%)	30 (12%)	80 (31%)	65 (25%)	54 (21%)
- Item 2	21 (8.2%)	75 (29%)	86 (34%)	55 (22%)	18 (7.1%)
- Item 12	34 (13%)	38 (15%)	76 (30%)	59 (23%)	48 (19%)
	Survey Mean (SD) Score		Interview Mean (SD) Score		
GBMMS: Total	32.9 (4.9)		31.1 (5.3)		
GBMMS: Lack of Support	9.7 (2.5)		9.4 (3.1)		
GBMMS: Suspicion	16.0 (4.3)		15.6 (5.7)		
GBMMS: Group Based Health Disparities	10.5 (2.6)		11.4 (3.6)		

**Table 3:** Total GBMMS and Subscale Multivariate Regression Outcomes

Variable	Beta	95% CI^1^	p-value
**Group Based Medical Mistrust (GBMM)**			
Age	−0.15	−0.21, −0.09	0.001***
Allopathic Provider Type (ref)	-	-	-
Holistic	1.3	−011,2.7	0.070
Both (Allopathic and Holistic)	1.9	0.53,3.3	0.007
Not Married	−2.3	−3.5, −1.1	0.001***
**Lack of Support (LOS)**			
Age	−0.06	−0.09, −0.03	0.001***
Has Children	−0.90	−1.6, −0.25	0.007***
Public health Insurance	−1.3	−2.0, −0.67	0.001***
Allopathic Provider Type (ref)	-	-	-
Holistic	.08	−0.61,0.77	0.8
Both (Allopathic and Holistic)	1.1	0.41,1.8	0.002***
**Suspicion (SUS)**			
Age	−0.19	−0.24, −0.14	0.001***
Education			
GED/HS	—	—	
College	0.15	−0.96, 1.3	0.8
Grad/Prof	2.0	0.72, 3.3	0.003***
Other	−0.53	−2.4, 1.4	0.6
Allopathic Provider Type (ref)	-	-	-
Holistic	1.3	0.14,2.4	0.027*
Both (Allopathic and Holistic)	1.8	0.60,3.0	0.003***
**Group Based Health Disparities (DISP)**			
Age	−0.06	−0.09, −0.03	0.001***
Education			
GED/HS	—	—	
College	0.43	−0.29, 1.2	0.2
Grad/Prof	1.7	0.81, 2.5	0.001***
Other	−0.13	−1.4,1.1	0.8
Has Children	−0.79	−1.4, −0.14	0.018*

1 CI = Confi dence Interval

* (p < 0.05);

*** (p < 0)

## Data Availability

The data that support the findings of this study are available on request from the corresponding author. The data are not publicly available due to privacy or ethical restrictions.

## Abbreviations

CVVP: Chronic Vulvovaginal Pain
IBWRH-SEM: Integrated Black Women’s Reproductive Health Social Ecological Model
GBMMS: Group Based Medical Mistrust Scale
SUS: Suspicion
LOS: lack of support
DISP: Group Disparities in Health Care
SEM: Social Ecological Model
PHCRP: Public Health Critical Race Praxis
BFT: Black Feminist Thought
RJ: Reproductive Justice
SDOH: Social Determinants of Health
CTSI: Clinical and Translational Science Institute
CAB: Community Advisory Board
SSI: Semi-Structured Interviews
